# Mycobacterium porcinum Disseminated Infection in Non-severely Immunocompromised Host

**DOI:** 10.7759/cureus.55889

**Published:** 2024-03-10

**Authors:** Shuva Shah, Kashaf Zaidi, Will Onyia

**Affiliations:** 1 Internal Medicine, AdventHealth Orlando, Orlando, USA; 2 Infectious Disease, AdventHealth Orlando, Orlando, USA

**Keywords:** nontuberculous mycobacteria (ntm), speciation, antibiotic susceptibility testing, mycobacterium porcinum, immuno-compromise

## Abstract

*Mycobacterium porcinum* is a nontuberculous mycobacteria (NTM) recently identified to cause human infection. Correct speciation of NTMs can be difficult and result in misdiagnosis and delayed treatment. Because of the paucity of the literature, there is a lack of awareness of the possibility of serious infections caused by *M. porcinum*. Although severe infections tend to occur in individuals with certain risk factors, the primary being an immunocompromised state, our case illustrates that it can also be possible in non-severely immunocompromised individuals.

A 65-year-old male with a medical history of diabetes mellitus (DM), end-stage renal disease (ESRD) on hemodialysis (HD), congestive heart failure (CHF), and chronic obstructive pulmonary disease (COPD) was admitted to the emergency room due to a laceration on his right lower leg following a fall. He reported shortness of breath but denied other respiratory symptoms. On examination, he showed signs of infection and increased oxygen requirement compared to baseline. Blood culture was positive for acid-fast bacilli (AFB), initially reported as *M. avium complex* (MAC) and later confirmed as *M. porcinum* through gene sequencing and morphology analysis. Interval blood cultures taken a week later confirmed true *M. porcinum* bacteremia. Treatment initially involved intravenous antibiotics- imipenem and ciprofloxacin before transitioning to oral linezolid and ciprofloxacin based on sensitivities. Following 10 days of antibiotic therapy, subsequent blood cultures returned negative, and treatment with oral antibiotics was advised, with continued outpatient follow-up with infectious disease in two weeks.

*M. porcinum*, typically considered a contaminant in healthy individuals, was identified as the causative agent of a disseminated infection in a non-severely immunocompromised patient. This case underscores the importance of accurately identifying the specific mycobacterial species, confirming true infection, and conducting antibiotic susceptibility testing due to the distinct antibiotic susceptibility profile of *M. porcinum* compared to other NTM like MAC.

## Introduction

Nontuberculous mycobacteria (NTM) have received increased attention as the incidence of infections caused by NTM is increasing worldwide. The rising incidence and prevalence of these infections are due to the increasing population of patients who are immunocompromised from primary disease, the use of immunosuppressive drugs, and the increasing number of patients using various types of medical devices for support [1,2].

NTMs are environmental and water-avid pathogens that cause a variety of infections. *Mycobacterium porcinum* is one of the NTM that was initially identified to cause submandibular lymphadenitis in swine and was first recognized to cause human infection in 2004 [3]. It has been commonly identified in localized cutaneous disease but also implicated in disseminated systemic infection [4]. Immunodeficiency is a significant risk factor for systemic disease for NTM. We found one case report of *M. porcinum* infection in the literature review. We present a rare case of disseminated *M. porcinum* infection and its pathology in a non-severely immunocompromised host. We aim to review relevant literature and compare it to our case.

## Case presentation

A 65-year-old male with an extensive past medical history, including diabetes mellitus (DM), end-stage renal disease (ESRD) on hemodialysis (HD), congestive heart failure (CHF), and chronic obstructive pulmonary disease (COPD), was brought to emergency from a skilled nursing facility (SNF) following a fall same day. The patient was a poor historian, and based on records, he lost his balance while trying to get up from a sitting position, had a fall, and sustained a laceration of the right lower leg. On review of the system, he endorsed shortness of breath and generalized weakness but denied cough, chest pain, and fever.

In the emergency room, he had a low-grade fever of 100.0F, tachycardia (HR, 121 bpm), and oxygen saturation of 96% on a 4 L nasal cannula (on chronic 3 L oxygen at baseline). Physical examination revealed two large skin tears (6 × 4 cm^2^ and 4 × 4 cm^2^) on the anterior aspect of the right lower extremity below the knee and rales in bilateral lower lung fields. The rest of the physical examination was within normal limits.

Among other investigations, two sets of routine peripheral blood cultures were sent on the day of admission (day 1). One of two blood cultures was positive for acid-fast bacilli on day 7, which was reported as a DNA probe positive for *M. avium* complex (MAC). It prompted further investigations like dedicated AFB blood culture (collected on day 8), HIV, and CT chest. HIV came out to be negative, and given the absence of immunosuppressive medication use and underlying immune deficiency syndrome, severe immunocompromised status was ruled out. CT chest showed nodular lung disease, which was thought to be likely MAC, given the blood culture results. A repeat AFB blood culture was collected on day 8, and AFB was collected on day 15 but had not been speciated yet. Induced sputum AFB cultures were sent on day 17. Pulmonology was consulted for bronchoscopy and BAL culture to confirm MAC infection. While the investigations were being done, the patient was started on therapy for MAC with azithromycin 500 mg oral once daily, ethambutol 15 mg/kg/day oral once daily, and rifabutin 300 mg oral once daily. Sputum AFB culture was positive for MAC. However, the bronchial secretion culture collected one day later (day 18) did not grow MAC. The likelihood of disseminated MAC was low, considering the absence of a severe immunocompromised state and negative BAL culture.

**Figure 1:**
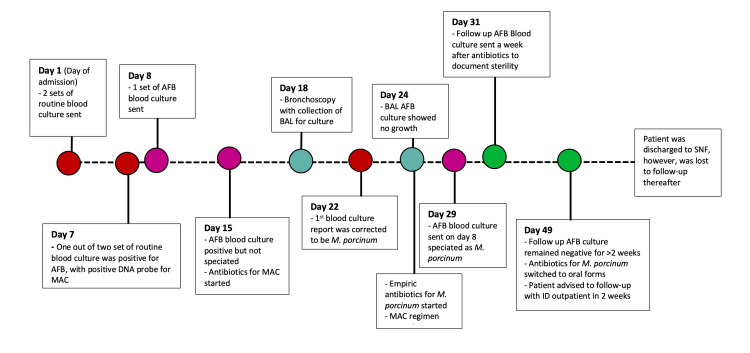
Timeline of investigations and antibiotics AFB, acid-fast bacilli; MAC, Mycobacterium avium complex, BAL, bronchoalveolar lavage; HD, hemodialysis; ID, infectious disease; SNF, skilled nursing facility

The peripheral blood culture collected on day 1 was initially reported to be MAC, which was later corrected to *M. porcinum* on day 22, confirmed by colonial morphology and beta subunit of RNA polymerase (rpoB) gene sequencing. The MAC DNA probe was also repeated, which came out to be negative. The question remained whether a single peripheral blood culture with *M. porcinum* represented true bacteremia and if it required treatment. This was answered by the second set of blood cultures collected on day 8, which grew *M. porcinum* on day 29, indicating true bacteremia. See Figure 1 for a timeline of investigations and antibiotics.

The patient was empirically started on intravenous (IV) imipenem 500 mg twice daily and oral ciprofloxacin 500 mg once daily. Antibiotic susceptibility test (AST) via a non-radiometric macrodilution assay revealed that the *M. porcinum* was susceptible to multiple antibiotics, including amikacin, cefoxitin, ciprofloxacin, moxifloxacin, imipenem, linezolid, tobramycin, and trimethoprim/sulfamethoxazole. See Table 1 for minimum inhibitory concentration (MIC data).

**Table 1:** Antibiotic susceptibility of Mycobacterium porcinum AST, antibiotic susceptibility test; MIC, minimum inhibitory concentration; S, susceptible; R, resistant; NI, no CLSI interpretive guideline for this antibiotic/organism combination

Antibiotics	MIC mcg/mL	Interpretation
Amikacin (IV)	≤8	S
Augmentin	32/18	NI
Azithromycin	128	NI
Azithromycin (day 14)	>258	NI
Cefepime	>32	NI
Cefotaxime	>64	NI
Cefoxitin	≤16	S
Ceftriaxone	>64	NI
Ciprofloxacin	≤1	S
Clarithromycin	8	R
Clarithromycin (day 14)	8	R
Clofazimine	≤0.5	NI
Clofazimine/amikacin	≤0.6/2	NI
Doxycycline	18	R
Gentamicin	≤2	NI
Imipenem	≤2	S
Kanamycin	≤8	NI
Linezolid	≤1	S
Minocycline	8	NI
Moxifloxacin	≤0.5	S
Tigecycline	≤0.25	NI
Tobramycin	≤2	S
Trimethoprim/sulfamethoxazole	≤0.5/9.5	S

Since empiric antibiotics were sensitive based on AST, they were continued during hospitalization. A repeat AFB blood culture after 10 days of antibiotics was negative. The patient was switched to oral antibiotics prior to discharge, including linezolid 600 mg twice daily and ciprofloxacin 500 mg once daily. At this point, the patient had received four weeks and five days of antibiotics and was discharged to an SNF with instructions to continue oral linezolid and ciprofloxacin and follow up with infectious disease outpatient in two weeks; however, he was lost to follow-up.

## Discussion

NTM contain more than 150 species and have been classified based on growth rate (slow growing takes more than seven days, and rapidly growing takes seven days or fewer to form colonies) and colony pigmentation [5-10]. They are environmental organisms, and about 25 species are pathogenic and cause the majority of clinically significant diseases in humans [6]. The most common pathogenic NTM encountered in clinical practice are MAC, *M. fortuitum*, *M. kansasii*, and others, including *M. abscessus* and *M. marinum* [10]. Identification of these various *Mycobacterium* strains from various clinical specimens has been enhanced lately by the availability of various molecular testing platforms. 

*M. porcinum* is rapidly growing mycobacteria (RGM), negative for pigmentation, and included in *M. fortuitum* complex (MFC) along with *M. fortuitum*, *M. peregrinum*, *M. septicum*, and eight other species [7,11]. *M. porcinum* shows regional variation, with the maximum number of isolates from Texas followed by Florida and other southern coastal states. It has been associated with outbreaks and pseudo-outbreaks, with the potential source being the community and hospital water system [12-14]. NTM are commonly associated with lung disease, while *M. porcinum* is mainly implicated in localized cutaneous disease, though disseminated systemic infections have also been identified.

Wallace et al. reported wound infections (62%), central catheter infections and/or bacteremia (16%), and possible pneumonitis (18%) as clinical infections caused by *M. porcinum* [4]. In the literature review, we found one case report of *M. porcinum* infection: peritonitis in continuous ambulatory peritoneal dialysis (CAPD) patients [9]. NTM are opportunistic pathogens, and factors known to predispose infection are structural lung diseases, like COPD, cystic fibrosis (CF), and prior tuberculosis, with disseminated infection seen in patients with severe immunocompromise, like HIV/AIDS, transplant recipients, hematologic malignancy, and inherited immune syndrome affecting IFN gamma and interleukin 12 and those on immunosuppressive therapy with TNF alpha inhibitors [5,10]. Living in the coastal area, the presence of an open skin wound, and underlying COPD were some of the factors predisposing our case to *M. porcinum* infection; however, no factors causing severe immunocompromise that would have predisposed to disseminated infection were identified. 

RGMs appear as gram-positive rods and may be mistaken as contaminants [8]. Even with a positive culture, there is the dilemma of true infection versus contamination and/or colonization. One study done on 14 *M. porcinum* isolates from CF patients reported no worsening of pulmonary symptoms, rather clinical improvement despite using antibiotics that did not target *M. porcinum* and no growth of *M. porcinum* in the majority of patients in post-discharge culture, suggesting it to be a transient colonizer than a pathogen [12]. This contrasts the outbreak reported by Brown-Elliott et al. with 50-55 isolates of *M. porcinum*, which were associated with clinical disease (infected central catheters, localized abscesses, and pulmonary infection) except for some isolates, which were respiratory contaminants [13].

Correct speciation of NTM is necessary as antimicrobial susceptibilities vary, which was another challenge we faced, as *M. porcinum* in blood culture was initially misidentified as MAC. MAC is the most identified NTM; it is slow-growing (takes seven days or more to form colonies) and known to cause pulmonary disease. There is ample focus on diagnosis and treatment guidelines on MAC by the American Thoracic Society (ATS) and the Infectious Diseases Society of America (IDSA), which is not the case for rare pathogens like *M. porcinum* [11]. Identification of MAC in blood leads to initiating antibiotics toward MAC and further testing like BAL to confirm infection and source. BAL culture was negative for both MAC and *M. porcinum*. 

Available methods of species identification include biochemical tests, DNA probes, high-performance liquid chromatography (HPLC), extended antibiotic in vitro susceptibility testing, DNA sequencing or polymerase chain reaction (PCR) restriction endonuclease assay, and matrix-assisted laser desorption ionization-time of flight (MALDI-TOF) mass spectrometry [10]. The initial identification of MAC in our patient was via DNA probe on day 7. There is evidence of cross-reactivity, especially among *M. fortuitum* group and probes directed to MAC, leading to misidentification of less frequently identified NTM species [15,16]. As our case illustrates, the correct identification of *M. porcinum* can be difficult or at least delayed. Regardless, many experts recommend identifying NTM at the species level as RGM are resistant to most first-line antitubercular medication and have different susceptibilities [7,17].

*M. porcinum* has been reported to be sensitive to fluoroquinolones, including ciprofloxacin, levofloxacin, gatifloxacin, and moxifloxacin, and cephalosporin, including cefoxitin, imipenem, amikacin, linezolid, sulfamethoxazole, and clarithromycin [4,7,9,18]. This is similar to the sensitivity pattern seen in our case except for resistance to clarithromycin. The erythromycin-inducible methylase (erm) gene can cause inducible resistance to macrolides in several NTM species. Due to a lack of adequate studies, although it is unclear if species other than *M. fortuitum* in MFC develop resistance, it is recommended by ATS guidelines to use macrolides with caution [11]. Regarding the duration of treatment, there are no specific recommendations for *M. porcinum*. ATS/IDSA guidelines recommend treatment for six to 12 months after immune restoration in disseminated infection by non-MAC species of NTM in non-HIV patients [10]. Reports on NTM peritonitis in CAPD recommend four to six weeks (uncomplicated cases) to six months of therapy [8,9].

## Conclusions

It is essential to know that rare pathogens like *M. porcinum* can cause disseminated disease in non-immunocompromised patients, in addition to skin and soft tissue infections, catheter-related infections, and pneumonitis. Difficulty in identifying organisms and recognizing them as potential pathogens may be part of the reason why it is not widely discussed, leading to a missed diagnosis. Fortunately, *M. porcinum* is easily treatable with sufficient data on antibiotic susceptibility. More case reports are needed to help form guidelines on diagnostic criteria and treatment duration.
